# Gene-specific sex effects on eosinophil infiltration in leishmaniasis

**DOI:** 10.1186/s13293-016-0117-3

**Published:** 2016-11-22

**Authors:** Martina Slapničková, Valeriya Volkova, Marie Čepičková, Tatyana Kobets, Matyáš Šíma, Milena Svobodová, Peter Demant, Marie Lipoldová

**Affiliations:** 1 Laboratory of Molecular and Cellular Immunology, Institute of Molecular Genetics, Academy of Sciences of the Czech Republic, Vídeňská 1083, 142 20 Prague, Czech Republic; 2 Faculty of Science, Charles University, 128 44 Prague, Czech Republic; 3 Roswell Park Cancer Institute, Buffalo, NY 14263 USA

**Keywords:** *Leishmania major*, Mouse model, Eosinophil infiltration, Genetic control, QTL, Sex influence

## Abstract

**Background:**

Sex influences susceptibility to many infectious diseases, including some manifestations of leishmaniasis. The disease is caused by parasites that enter to the skin and can spread to the lymph nodes, spleen, liver, bone marrow, and sometimes lungs. Parasites induce host defenses including cell infiltration, leading to protective or ineffective inflammation. These responses are often influenced by host genotype and sex. We analyzed the role of sex in the impact of specific gene loci on eosinophil infiltration and its functional relevance.

**Methods:**

We studied the genetic control of infiltration of eosinophils into the inguinal lymph nodes after 8 weeks of *Leishmania major* infection using mouse strains BALB/c, STS, and recombinant congenic strains CcS-1,-3,-4,-5,-7,-9,-11,-12,-15,-16,-18, and -20, each of which contains a different random set of 12.5% genes from the parental “donor” strain STS and 87.5% genes from the “background” strain BALB/c. Numbers of eosinophils were counted in hematoxylin-eosin-stained sections of the inguinal lymph nodes under a light microscope. Parasite load was determined using PCR-ELISA.

**Results:**

The lymph nodes of resistant STS and susceptible BALB/c mice contained very low and intermediate numbers of eosinophils, respectively. Unexpectedly, eosinophil infiltration in strain CcS-9 exceeded that in BALB/c and STS and was higher in males than in females. We searched for genes controlling high eosinophil infiltration in CcS-9 mice by linkage analysis in F_2_ hybrids between BALB/c and CcS-9 and detected four loci controlling eosinophil numbers. *Lmr14* (chromosome 2) and *Lmr25* (chromosome 5) operate independently from other genes (main effects). *Lmr14* functions only in males, the effect of *Lmr25* is sex independent. *Lmr15* (chromosome 11) and *Lmr26* (chromosome 9) operate in cooperation (non-additive interaction) with each other. This interaction was significant in males only, but sex-marker interaction was not significant. Eosinophil infiltration was positively correlated with parasite load in lymph nodes of F_2_ hybrids in males, but not in females.

**Conclusions:**

We demonstrated a strong influence of sex on numbers of eosinophils in the lymph nodes after *L. major* infection and present the first identification of sex-dependent autosomal loci controlling eosinophilic infiltration. The positive correlation between eosinophil infiltration and parasite load in males suggests that this sex-dependent eosinophilic infiltration reflects ineffective inflammation.

## Background

Sex influences susceptibility to many infectious diseases [1], including some manifestations of leishmaniasis [2], a disease that threatens several hundred million people in 98 countries [3]. Disability-adjusted life years (DALYs) due to leishmaniasis are globally increasing [4]. The disease is caused by intracellular protozoan parasites of the genus *Leishmania* and is transmitted to the vertebrates by the bite of female phlebotomine sand flies.

*Leishmania* parasites infect the so-called professional phagocytes (neutrophils, monocytes, and macrophages), as well as dendritic cells and fibroblasts. The major host cell is the macrophage, where parasites multiply, eventually rupturing the cell and spread to the uninfected cells (reviewed in [5]). Infected monocytes and macrophages circulating in the peripheral blood are believed to be carriers of the parasite to distal sites [6]. In the dermis, parasites cause the cutaneous form of the disease (which can be localized or diffuse), whereas infection of the mucosa gives rise to mucocutaneous leishmaniasis. The metastatic spread of the infection to the spleen and liver results in visceral leishmaniasis. Although these are the major sites of visceral disease, parasites can also enter other organs, such as the bone marrow, lymph nodes, and lungs (reviewed in [5]). Presence of parasites in organs usually induces inflammation through cascade of signals that leads to recruitment of inflammatory cells, such as neutrophils, macrophages, eosinophils, and dendritic cells. These innate immune cells might phagocytose parasites and/or produce cytokines and chemokines that activate both innate and adaptive immune responses. Resulting responses can be protective and eliminate parasites, or ineffective and lead to chronic inflammation [7].

The sex of the host influences the incidence of disease, parasite burden, pathology, mortality, and immunological response against various parasites, including *Leishmania* both in humans and in rodents (reviewed in [7–12]).

In general, sex bias is observed after infection with *Leishmania* parasites, and men are more frequently infected than women ([13–15]; reviewed in [11, 12]), although in certain areas no sex bias in prevalence of disease was observed [16]. The higher susceptibility of males also applies to hamster [17] and mouse [18, 19]; reviewed in [12] models of leishmaniasis. The effect of male orchidectomy and female testosterone replacement studies suggests that the hormone testosterone can modulate systemic *L. major* infection in BALB/cAnPt, DBA/2N, DBA/2J, and F_1_ hybrids (BALB/cAnPt x DBA/2N) mouse strains [18].

Importantly, the host genes, including those regulated differently in males and females, play a significant role in determining susceptibility and organ tropism for infectious diseases. Experimental data have shown different sex influence on susceptibility to relatively closely related pathogen species [20, 21], different sex biases in susceptibility to the same *Leishmania* species in different host genotypes [21, 22], and different sex and genetic influence on organ-specific pathology [21, 23, 24]. For example, high resistance to skin lesions induced by *L. mexicana* was observed in females but not in males of DBA/2 mice, but the sex effect was opposite in *L. major* infection [20].

Genotype influence on sex differences was defined in the studies of *L. major* infection [22, 24]. Giannini [22] found no sex effect on *L. major*-induced skin pathology and mortality in BALB/cJ mice, but a higher susceptibility of B10.129(10M)ScSn females than males. The comparison of *L. major* susceptibility in two strains, BALB/cHeA and CcS-11 [24], has shown that there is no significant sex influence on skin lesion development, splenomegaly, and hepatomegaly in these strains. However, parasite numbers in lymph nodes are higher in both BALB/c and CcS-11 males; moreover, CcS-11 males have higher parasite load in spleens, showing an organ-specific, sex-, and genotype-dependent pathology [24].

In the present study, we address influence of genotype and sex on infiltration of eosinophil leukocytes into the inguinal lymph nodes of *L. major*-infected mice. Eosinophils are granulocytes that develop in the bone marrow from pluripotent progenitors. They are released into the peripheral blood in phenotypically mature state and can be activated and recruited into tissues in response to appropriate stimuli, most notable IL-5, and the eotaxin chemokines [25].

Eosinophils contribute to the initiation of inflammatory and adaptive responses due to their bidirectional interactions with dendritic cells and T cells, as well as their large spectrum of secreted cytokines and soluble mediators. They have key immunoregulatory roles as professional antigen-presenting cells and modulators of functions of CD4^+^ T cells, dendritic cells, B cells, mast cells, neutrophils, and basophils [26].

Eosinophil-associated disorders can affect practically all tissues and organs in the body, either individually or in combination. They are involved in inflammatory conditions affecting the skin, cardiovascular, nervous and renal system, gastrointestinal tract, and upper and lower airways [27, 28], are key effector cells in eosinophilic asthma [29], and their interaction with peripheral nerves has impact on pathology of many diseases. In addition, they are also involved in regulatory mechanisms modulating local and systemic immune responses and remodeling and repair mechanisms [30].

Eosinophils may have an important role in maintaining host survival in life-threatening viral infections [31]. They combat worms such as *Angiostrongylus cantonensis* [32], *Nippostrongylus brasiliensis* [33], *Litomosoides sigmodontis* [34], and *Brugia pahangi* [35]; but their role in response to other nematoda is more complex. Eosinophils have no role in protection against *Schistosoma mansoni* [36]. They even promote larval growth in primary infection with *Trichinella spiralis* [37], but they mediate protective immunity against secondary infection with this nematode [38].

Activated eosinophils can kill [39] or support killing of *L. major* parasites [40]; however, in chronic disease, eosinophil infiltration might be a consequence of an ineffective elimination of these parasites and/or an excessive inflammatory response to the present pathogens [41].

Here, we analyzed genetic influence on eosinophil infiltration after *L. major* infection into the lymph nodes of strains BALB/cHeA (BALB/c), STS/A (STS), and selected 12 (out of 20) RC strains of CcS/Dem series [42]. Each of the 20 RC CcS/Dem strains contains a different unique set of approximately 12.5% genes of the donor strain STS on the genetic background of BALB/c. We found surprisingly high numbers of eosinophils in the inguinal lymph nodes of the strain CcS-9, males containing higher numbers of eosinophils than females. We analyzed genetics of this infiltration using microsatellite DNA markers and mapped four loci that control eosinophil numbers after *L. major* infection, one of them being strongly influenced by sex. We also found that the numbers of eosinophils in the lymph nodes correlate positively with the parasite load and that this correlation is partly genetically controlled and is higher in males than in females.

## Methods

### Mice

Tests of strain differences in eosinophil infiltration: Mice of the strains BALB/c (27 females, 27 males), STS (8 females, 9 males), CcS-1 (10 females, 13 males), CcS-3 (10 females, 10 males), CcS-4 (13 females, 12 males), CcS-5 (19 females, 27 males), CcS-7 (8 females, 12 males), CcS-9 (15 females, 10 males), CcS-11 (13 females, 13 males), CcS-12 (16 females, 12 males), CcS-15 (7 females, 12 males), CcS-16 (10 females, 13 males), CcS-18 (5 females, 3 males), and CcS-20 (13 females, 18 males) were infected with *L. major* as described previously [43, 44]. Mice were tested in eight successive experimental groups and were euthanized 8 weeks after infection. The age of mice at the time of infection was 7 to 47 weeks (mean 15 weeks, median 14 weeks).

A linkage study of eosinophil infiltration: F_2_ hybrids between CcS-9 and BALB/c (age 11 to 21 weeks at the time of infection, mean and median age 14.8 and 15 weeks, respectively) were produced at the Institute of Molecular Genetics. When used for these experiments, the CcS-9 was in the 40th generation of inbreeding and therefore highly homozygous. Two hundred fifty-four F_2_ hybrids between BALB/c and CcS-9 comprised 139 females and 115 males. Mice of the background parental strains BALB/c (18 females, 17 males) and STS (8 females, 6 males) and the RC strain CcS-9 (16 females, 14 males), 7 to 20 weeks old at the time of infection (mean 13 weeks, median 13 weeks), were used as controls. During the experiment, male and female mice were placed into separate rooms and males were caged individually. F_2_ mice were tested in three independent experimental groups.

### Ethical statement

All experimental procedures in this study comply with the Czech Government Requirements under the Policy of Animal Protection Law (No.246/1992) and with the regulations of the Ministry of Agriculture of the Czech Republic (No.207/2004), which are in agreement with all relevant European Union guidelines for work with animals and were approved by the Institutional Animal Care Committee of the Institute of Molecular Genetics AS CR and by Departmental Expert Committee for the Approval of Projects of Experiments on Animals of the Academy of Sciences of the Czech Republic (permissions Nr. 274/2011; 89/2013).

### Parasites

*L. major* LV 561 (MHOM/IL/67/LRC-L137 JERICHO II) was maintained in rump lesions of BALB/c females. Amastigotes were transformed to promastigotes using SNB-9 [43]. 10^7^ promastigotes from the passage, two cultivated for 6 days were inoculated in 50 μl sterile saline s.c. into mouse rump [44].

### Disease phenotype

The size of the primary skin lesion was measured weekly using a Vernier caliper gauge. The mice were killed 8 weeks after infection and inguinal lymph nodes draining the site of infection were collected for further analysis.

### Histological analysis

Inguinal lymph nodes of female and male mice were fixed in 10% neutral buffered formalin (NBF; approximately 4% formaldehyde) and embedded in paraffin using automatic tissue processor. Tissue sections (5–7 μm) were stained with hematoxylin, differentiated into 1% acid alcohol, stained with 1% alcoholic eosin, dehydrated, assembled with permanent mounting medium, and analyzed under a light microscope (Olympus BX51; Olympus Optical Co. (EUROPA) GMBH., Hamburg, Germany).

Eosinophil infiltration in the experiment with parental strains BALB/c, STS, and 12 RC strains was assessed using a semi-quantitative scoring system: 0, no eosinophil; 0.25, 1 eosinophil; 0.5, 2 eosinophils; 0.75, 3–4 eosinophils; 1, 5 eosinophils; 1.5, 6 eosinophils; 2, 7 eosinophils; 2.5, 8–9 eosinophils; 3, 10–15 eosinophils; and 4, more than 15 eosinophils per lymph node section (one section was used in experiment  with parental strains BALB/c, STS and 12 RC strains).

In F_2_ mice, as well as the parental strains BALB, STS, and CcS-9, eosinophil numbers were determined quantitatively. The total number of eosinophils was counted in the node section and each lymph node was assessed in four independent sections. The mean value of these four counts was used to calculate the role of genetic factors in control of eosinophil infiltration. Sixty slides from 15 mice were blindly recounted by an independent investigator with concordant results (*R* = 0.913, *P* value = 5.66 × 10^−29^).

### Genotyping of F_2_ mice by PCR

DNA was isolated from tails using a standard proteinase procedure. The strain CcS-9 differs from BALB/c at STS-derived segments on eight chromosomes ([45] and unpublished results). These differential segments were typed in the F_2_ hybrid mice between CcS-9 and BALB/c using 18 microsatellite markers (Research Genetics, Huntsville, FL, USA): D2Mit283, D2Mit148, D4Mit172, D4Mit23, D4Mit53, D4Mit17, D5Mit24, D5Mit143, D6Mit122, D6Mit274, D9Mit15, D11Mit141, D11Mit242, D11Nds18, D11Nds10, D16Mit19, D17Mit120, and D17Mit122. The markers were selected because their genomic location makes them suitable to detect linkage. The maximum distance between any two markers in the chromosomal segments derived from the strain STS or from the nearest BALB/c derived markers was 12.46 cM, and mean distance was 4.67 cM. The PCR genotyping for markers with fragment length difference more than 8 bp was performed using unlabeled primers as in [46, 47]. The PCR genotyping for markers with fragment length difference less than 8 bp was performed using [γ-^32^P]ATP end-labeled primers as described elsewhere [48].

### Measurement of parasite load in lymph nodes

Total DNA was isolated from the frozen lymph nodes, and parasite load was measured using PCR-ELISA according to the previously published protocol [49]. Briefly, for detection of *Leishmania* parasite DNA, in total DNA, PCR was performed using two primers (digoxigenin-labeled F 5′-ATT TTA CAC CAA CCC CCA GTT-3′ and biotin-labeled R 5′-GTG GGG GAG GGG CGT TCT-3′ (VBC Genomics Biosciences Research, Austria). The 120-bp fragment within the conserved region of the kinetoplast minicircle of *Leishmania* parasite was amplified. In each PCR reaction, 50 ng of extracted total DNA was used. As a positive control, 20 ng of *L. major* DNA per reaction was amplified as a highest concentration of the standard. A 26-cycle PCR reaction was used for quantification of parasites. Parasite load was determined by measurement of the PCR product with the modified ELISA protocol (Pharmingen, San Diego, USA). The concentration of *Leishmania* DNA was measured at the ELISA Reader Tecan with the curve fitter program KIM-E (Schoeller Pharma, Prague, Czech Republic) using least squares-based linear regression analysis [24, 49].

### Statistical analysis

The differences among BALB/c, STS, and CcS/Dem strains in eosinophil numbers in lymph nodes were evaluated by the analysis of variance (ANOVA) and Newman-Keuls multiple comparison test at 95% significance using the program Statistica for Windows 12.0 (StatSoft, Inc., Tulsa, OK, USA).

Differences between sexes in BALB/c, STS, and CcS/Dem strains were calculated by ANOVA (Statistica for Windows 12.0; StatSoft, Inc., Tulsa, OK, USA).

The role of genetic factors in control of eosinophil infiltration in F_2_ hybrids was examined by ANOVA (Statistica for Windows 12.0; StatSoft, Inc., Tulsa, OK, USA). In order to obtain normal distribution of the analyzed parameter required for ANOVA, the obtained values were transformed as shown in the legends of tables. Markers and interactions with *P* < 0.05 were combined in a single comparison. In all ANOVA analyses strain or genotype, sex, and age were fixed factors, and the experiment was considered a random parameter.

For each independent variable, the partial *R* ^2^ was computed in the usual way by subtracting the regression sums of squares of the model without the variable (SS(b1,b2,b3,b4|b0)) of interest from the regression sums of squares of the full model (SS(b1,b2,b3,b4,b5|b0)); this difference divided by total regression sums of squares ((SS(b1,b2,b3,b4,b5|b0)):

$$ \frac{\left(\mathrm{S}\mathrm{S}\left(\mathrm{b}1,\mathrm{b}2,\mathrm{b}3,\mathrm{b}4\Big|\mathrm{b}0\right)\right)\kern0.5em -\kern0.5em \left(\mathrm{S}\mathrm{S}\left(\mathrm{b}1,\mathrm{b}2,\mathrm{b}3,\mathrm{b}4,\mathrm{b}5\Big|\mathrm{b}0\right)\right)}{\Big(\mathrm{S}\mathrm{S}\left(\mathrm{b}1,\mathrm{b}2,\mathrm{b}3,\mathrm{b}4,\mathrm{b}5\Big|\mathrm{b}0\right)} $$

indicated the contribution of the independent variable.

To obtain whole-genome significance values (corrected *P* values) the observed *P* values (aT) were adjusted according to Lander and Schork [50] using the formula:

$$ {\upalpha}_{\mathrm{T}}*\approx \kern0.5em \left[C+\kern0.5em 2\rho Gh(T)\right]{\upalpha}_{\mathrm{T}} $$

where *G* = 1.75 Morgan (the length of the segregating part of the genome: 12.5% of 14 M); *C* = 8 (number of chromosomes segregating in cross between CcS-9 and BALB/c); *ρ* = 1.5 for F_2_ hybrids; *h*(*T*) = the observed statistics (F ratio).

The Spearman correlation coefficients between parasite numbers and eosinophil infiltration in the lymph nodes of F_2_ hybrid mice were computed using the program Statistica for Windows 12.0 (StatSoft, Inc., Tulsa, OK, USA).

## Results

### Infiltration of eosinophils into the inguinal lymph nodes in parental strains BALB/c and STS and selected RC strains

We infected with *L. major* both females and males of the strains BALB/c, STS, and RC strains CcS-1, CcS-3, CcS-4, CcS-5, CcS-7, CcS-9, CcS-11, CcS-12, CcS-15, CcS-16, CcS-18, and CcS-20 and used semi-quantitative scoring system to assess eosinophil infiltration (Table 1).

**Table 1:** Eosinophil numbers in inguinal lymph nodes of *L. major*-infected mice

		% of mice with number of eosinophils (graded as 0–4) in section of inguinal lymph node									
		0	0.25	0.5	0.75	1	1.5	2	2.5	3	4
Strain	Sex	0	1	2	3–4	5	6	7	8–9	10–15	>15
BALB/c	Females	100.00	0	0	0	0	0	0	0	0	0
	Males	81.48	0	3.70	0	*3.70*	*3.70*	*7.41*	0	0	0
STS	Females	100.00	0	0	0	0	0	0	0	0	0
	Males	100.00	0	0	0	0	0	0	0	0	0
CcS-1	Females	80.00	0	0	0	*10.00*	0	*10.00*	0	0	0
	Males	76.92	*7.69*	0	0	*15.38*	0	0	0	0	0
CcS-3	Females	100.00	0	0	0	0	0	0	0	0	0
	Males	90.00	0	0	0	*10.00*	0	0	0	0	0
CcS-4	Females	92.31	0	*7.69*	0	0	0	0	0	0	0
	Males	91.67	0	0	0	0	0	0	0	*8.33*	0
CcS-5	Females	100.00	0	0	0	0	0	0	0	0	0
	Males	96.30	0	0	0	*3.70*	0	0	0	0	0
CcS-7	Females	100.00	0	0	0	0	0	0	0	0	0
	Males	75.00	0	0	0	*25.00*	0	0	0	0	0
CcS-9	Females	*73.33*	0	0	0	*20.00*	0	*6.67*	0	0	0
	Males	*20.00*	0	*10.00*	0	*20.00*	0	*10.00*	*20.00*	*20.00*	0
CcS-11	Females	*84.62*	0	*7.69*	*7.69*	0	0	0	0	0	0
	Males	*61.54*	0	*23.08*	0	*7.69*	0	*7.69*	0	0	0
CcS-12	Females	*56.25*	0	0	0	*31.25*	*6.25*	0	0	*6.25*	0
	Males	*50.00*	0	*8.33*	0	*16.67*	0	*16.67*	0	0	*8.33*
CcS-15	Females	100.00	0	0	0	0	0	0	0	0	0
	Males	91.67	0	8.33	0	0	0	0	0	0	0
CcS-16	Females	90.00	0	10.00	0	0	0	0	0	0	0
	Males	100.00	0	0	0	0	0	0	0	0	0
CcS-18	Females	100.00	0	0	0	0	0	0	0	0	0
	Males	*33.33*	0	*66.67*	0	0	0	0	0	0	0
CcS-20	Females	92.31	0	0	0	*7.69*	0	0	0	0	0
	Males	95.00	0	0	0	*5.00*	0	0	0	0	0

Eosinophil numbers in lymph nodes depending on genotype and sex. Eosinophil infiltration was evaluated as described in the “Methods” section. Numbers higher than 75% are shown in italics

**Fig. 1:**
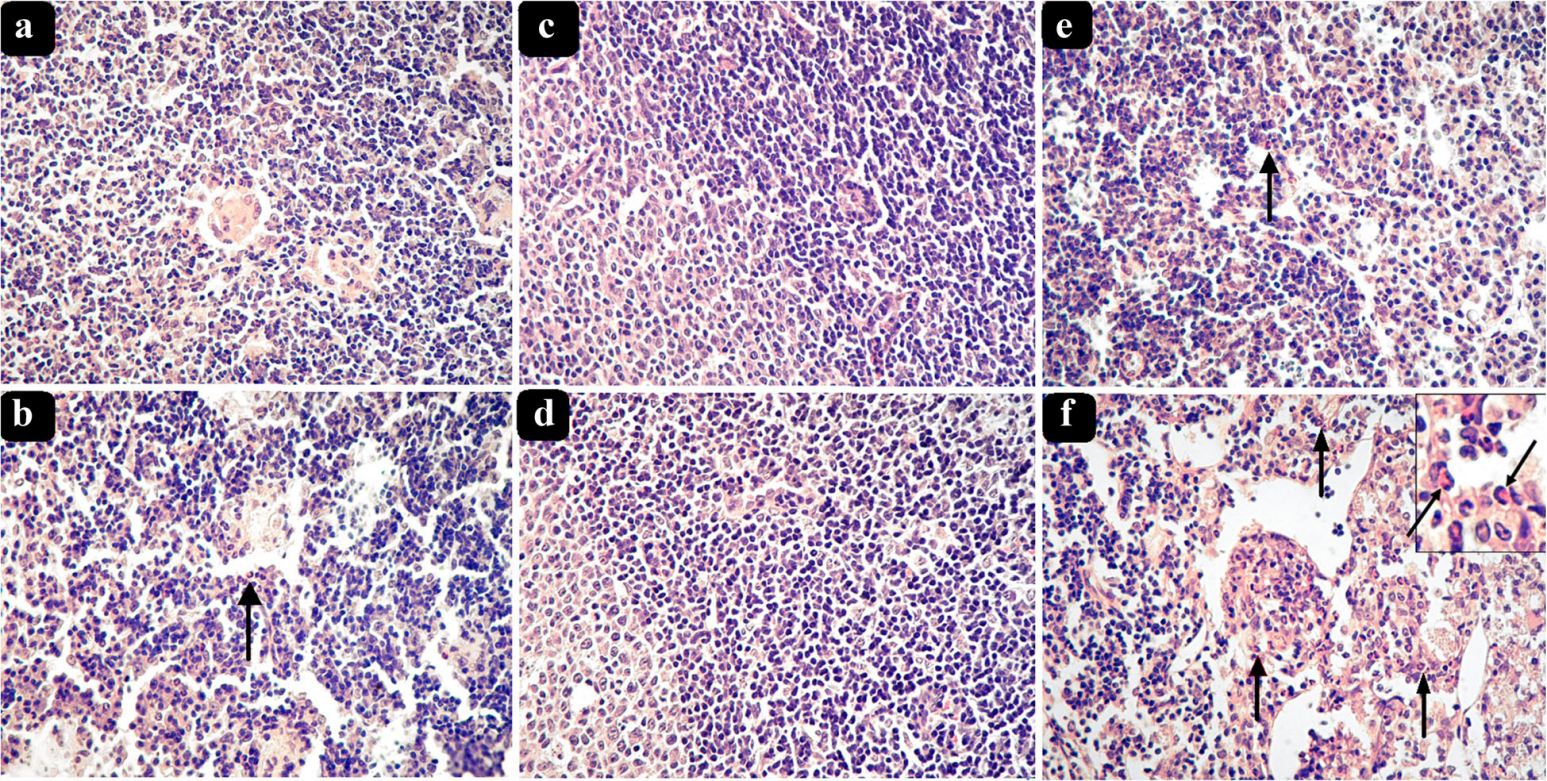
Eosinophils in hematoxylin-eosin-stained inguinal lymph node sections of *L. major-*infected female and male mice. **a** BALB/c female, **b** BALB/c male, **c** STS female, **d** STS male, **e** CcS-9 female, and **f** CcS-9 male with detail of eosinophils. *Arrows* show positions of eosinophils

These studies showed mild and no infiltration into the lymph nodes of parental strains BALB/c (Fig. 1a, b) and STS (Fig. 1c, d), respectively. Strains CcS-9 (*P* = 0.00020) (Fig. 1e, f) and CcS-12 (*P* = 0.0024) exhibit significantly higher eosinophil infiltration in their lymph nodes than the background parental strain BALB/c. BALB/c and CcS-9 males presented higher eosinophil infiltration than females of these strains *P* = 0.0089 and *P* = 0.016, respectively. 80% of examined CcS-9 males in comparison with 26.67% of CcS-9 females contained infiltrating eosinophils, 50% of males having 7 and more eosinophils in their lymph nodes (Table 1). Sex difference in strains CcS-7,-11, and -18 was not significant. Strain CcS-9 with the highest eosinophil infiltration (Table 1) was selected for further genetic studies.

### Four novel loci control eosinophil infiltration in leishmaniasis

We examined eosinophil numbers in lymph nodes in 254 F_2_ hybrids between the strains BALB/c and CcS-9. The strain CcS-9 differs from BALB/c at STS-derived genetic regions located at eight chromosomes ([45], Šíma unpublished data). These differential STS-derived segments were genotyped in the F_2_ hybrid mice using 18 microsatellite markers. A statistical analysis of linkage revealed four genetic loci that influence eosinophil infiltration into the inguinal lymph nodes after *L. major* infection.

**Fig. 2:**
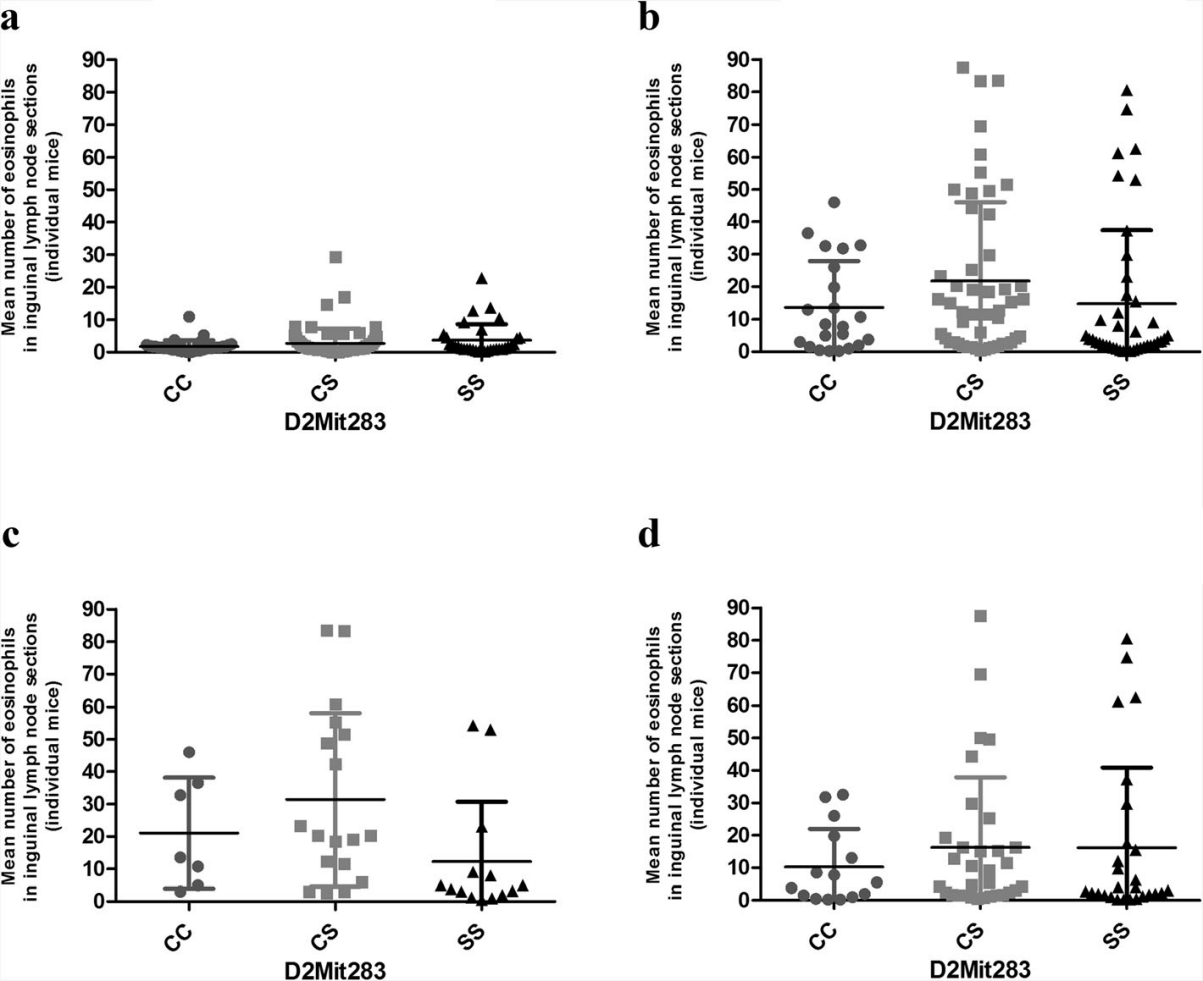
Effects of genotype and sex on eosinophil infiltration at *Lmr14* (D2Mit283) **a** females (corr. *P* = NS), **b** males (corr. *P* = NS), **c** males (CcS-9 × BALB/c)F_2_ cross (corr. *P* = 8.11 × 10^−3^), and **d** males (BALB/c × CcS-9)F_2_ cross (corr. *P* = NS).These data are shown for sex and genotype CC—BALB/c homozygotes, CS—heterozygotes, SS—STS homozygotes as mean ± SD. *NS* not significant

The effects of *Lmr14* (*L. major* response 14) linked to D2Mit283 (corrected *P* value = 0.0081) and *Lmr25* linked to D5Mit143 (corrected *P* value = 0.044) were detectable and significantly independent of each other or other genes (the main effects) (Table 2). *Lmr14* operated only in males (corr. *P* value of marker and sex interaction =0.0085)(Table 2, Fig. 2), higher numbers of eosinophils were associated with presence of BALB/c (C) allele (Fig. 2c). The *P* value for *Lmr14* was significant only in cross (CcS-9 × BALB/c)F_2_ (where the mother of the F_1_ hybrids was CcS-9 and the father was BALB/c) (Fig. 2c), but not in cross (BALB/c × CcS-9)F_2_ (where the mother was BALB/c and the father was CcS-9) (Fig. 2d). However, interaction between the cross and marker D2Mit283 was not significant (corr. *P* = 0.6). The effect of *Lmr25* was not influenced by sex, and higher numbers of eosinophils were observed in heterozygotes (Table 2).

**Table 2:** Main effect of loci that control eosinophil numbers in the inguinal lymph nodes of *L. major*-infected F_2_ hybrids between CcS-9 and BALB/c

Locus	Group	Marker	Genotype									*P* value	Corr. *P* value	% of expl. var.
			CC			CS			SS					
**Lmr14**	Both sexes	D2Mit283	**2.62**	1.229	±0.003	**3.62**	1.234	±0.002	**2.67**	1.229	±0.002	NS	NS	NA
				(*n* = 57)			(*n* = 111)			(*n* = 74)				
	Females		**1.24**	1.217	±0.003	**1.45**	1.220	±0.002	**1.62**	1.221	±0.003	NS	NS	NA
				(*n* = 35)			(*n* = 61)			(*n* = 32)				
	Males both crosses		**5.15**	1.239	±0.005	**8.94**	1.247	±0.003	**4.02**	1.236	±0.004	5.5 × 10^−2^	NS	NA
				(*n* = 22)			(*n* = 50)			(*n* = 41)				
	Males (BALB/c × CcS-9)F_2_		**3.37**	1.233	±0.006	**9.09**	1.242	±0.004	**3.78**	1.235	±0.005	NS	NS	NA
				(*n* = 15)			(*n* = 32)			(*n* = 27)				
	Males (CcS-9 × BALB/c)F_2_		**13.63**	1.253	±0.005	**18.34**	1.257	±0.004	**4.51**	1.237	±0.005	1.08 × 10^−4^	**8.11 × 10** ^**−3**^	**36.22**
				(*n* = 7)			(*n* = 18)			(*n* = 14)				
**Lmr25**	Both sexes	D5Mit143	**2.07**	1.225	±0.003	**4.33**	1.237	±0.002	**1.96**	1.225	±0.003	9.53 × 10^−4^	**4.36 × 10** ^**−2**^	**5.02**
				(*n* = 66)			(*n* = 107)			(*n* = 67)				
	Females		**1.09**	1.215	±0.003	**2.04**	1.225	±0.002	**1.20**	1.216	±0.003	6.3 × 10^−3^	NS	NA
				(*n* = 37)			(*n* = 56)			(*n* = 36)				
	Males		**5.71**	1.241	±0.004	**7.40**	1.244	±0.003	**4.41**	1.237	±0.004	NS	NS	NA
				(*n* = 31)			(*n* = 51)			(*n* = 31)				

Mean and SE values were obtained by analysis of variance. In order to obtain normal distribution required for analysis of variance, the value of eosinophil numbers in the inguinal lymph nodes was transformed by using the 0.1th power of natural logarithm of the (observed value ×1000). The numbers in bold give the average non-transformed values. C and S indicate the presence of BALB/c and STS allele, respectively

*n* number of mice

In contrast to the main effects of *Lmr14* and *Lmr25*, *Lmr15* (linked to D11Nds10) and *Lmr26* (linked to D9Mit15) operated in cooperation with each other (non-additive, epistatic, interaction) (corrected *P* = 0.010). F_2_ male mice of the cross (BALB/c × CcS-9)F_2_ with homozygous BALB/c (CC) alleles at both *Lmr26* and *Lmr15* had nearly nine times higher numbers of eosinophils in the lymph nodes than mice with homozygous STS (SS) alleles at both these loci, and nearly 90 times higher than mice with homozygous CC alleles at *Lmr26* and CS alleles at *Lmr15* (Table 3). The linkage was detected only in males, but the interaction between sex and marker was not significant (corr. *P* = 0.19).

**Table 3:** Interaction between loci controlling eosinophil numbers in the inguinal lymph nodes in *L. major*-infected F_2_ hybrids between CcS-9 and BALB/c

		D9Mit15 (**Lmr26**)								
		CC			CS			SS		
		*P* = 3.7 × 10^−2^			Corr. *P* = NS			% of expl. var. = NA		
D11Nds10 (**Lmr15**)	CC	**4.24**	1.236	±0.006	**3.03**	1.231	±0.004	**2.19**	1.226	±0.005
Both sexes				(*n* = 13)			(*n* = 26)			(*n* = 17)
	CS	**1.45**	1.220	±0.004	**3.76**	1.235	±0.003	**3.88**	1.235	±0.003
				(*n* = 21)			(*n* = 63)			(*n* = 39)
	SS	**2.56**	1.229	±0.005	**2.37**	1.228	±0.004	**3.36**	1.233	±0.005
				(*n* = 17)			(*n* = 30)			(*n* = 16)
		*P* = 0.67			Corr. *P* = NS			% of expl. var. = NA		
D11Nds10 (**Lmr15**)	CC	**1.89**	1.224	±0.007	**1.60**	1.221	±0.004	**1.42**	1.219	±0.005
Females				(*n* = 6)			(*n* = 18)			(*n* = 10)
Both crosses	CS	**1.37**	1.219	±0.005	**1.53**	1.220	±0.003	**2.28**	1.227	±0.005
				(*n* = 11)			(*n* = 38)			(*n* = 17)
	SS	**1.67**	1.222	±0.007	**1.46**	1.220	±0.004	**1.06**	1.214	±0.007
				(*n* = 7)			(*n* = 16)			(*n* = 6)
		*P* = 2.63 × 10^−4^			Corr. *P* = **1.037** × **10** ^**−2**^			% of expl. var. = **15.35**		
D11Nds10 (**Lmr15**)	CC	**11.71**	1.251	±0.010	**12.05**	1.251	±0.008	**3.49**	1.234	±0.008
Males				(*n* = 7)			(*n* = 8)			(*n* = 7)
Both crosses	CS	**1.06**	1.214	±0.007	**23.15**	1.260	±0.005	**12.22**	1.251	±0.004
				(*n* = 10)			(*n* = 25)			(*n* = 22)
	SS	**6.55**	1.243	±0.008	**5.18**	1.239	±0.005	**7.27**	1.244	±0.007
				(*n* = 10)			(*n* = 14)			(*n* = 10)
		*P* = 0.41			Corr. *P* = NS			% of expl. var. = NA		
D11Nds10 **(Lmr15)**	CC	**10.37**	1.249	±0.008	**12.60**	1.252	±0.006	**6.78**	1.243	±0.008
Males				(*n* = 2)			(*n* = 2)			(*n* = 3)
Cross CcS-9 × BALB	CS	**8.49**	1.246	±0.022	**21.20**	1.258	±0.004	**9.85**	1.248	±0.007
				(*n* = 2)			(*n* = 10)			(*n* = 10)
	SS	**6.92**	1.244	±0.012	**3.75**	1.235	±0.007	**13.88**	1.253	±0.022
				(*n* = 4)			(*n* = 4)			(*n* = 2)
		*P* = 3.97 × 10^−4^			Corr. *P* = *1.629* × **10** ^**-2**^			% of expl. var. = **21.19**		
D11Nds10 (**Lmr15**)	CC	**44.01**	1.267	±0.012	**32.78**	1.264	±0.010	**8.88**	1.247	±0.016
Males				(*n* = 5)			(*n* = 6)			(*n* = 4)
Cross BALB × CcS-9	CS	**0.47**	1.199	±0.010	**13.04**	1.252	±0.006	**12.40**	1.252	±0.006
				(*n* = 8)			(*n* = 15)			(*n* = 12)
	SS	**9.73**	1.248	±0.011	**6.78**	1.243	±0.007	**4.84**	1.238	±0.008
				(*n* = 6)			(*n* = 10)			(*n* = 8)

Mean and SE values were obtained by analysis of variance. In order to obtain normal distribution required for analysis of variance value of eosinophil numbers in serum inguinal lymph nodes was transformed by using the 0.1th power of natural logarithm of the (observed value ×1000). The numbers in bold give the average non-transformed values. C and S indicate the presence of BALB/c and STS allele, respectively

*n* number of mice

### Positive correlation between parasite numbers and eosinophils in the inguinal lymph nodes

We have determined parasite load in the lymph nodes of the F_2_ hybrids between BALB/c and CcS-9 and analyzed the relationship between parasite numbers in lymph nodes and eosinophil infiltration to this organ. In both sexes pooled, there was a positive correlation between parasite numbers and eosinophil infiltration *R* = 0.39, *P* = 1.3 × 10^−10^, and the correlation was significant in males *R* = 0.29, *P* = 0.0017, but not in females *R* = 0.14, *P* = 0.10. This correlation is at least partly controlled by *Lmr* loci, because in F_2_ hybrid mice, this correlation was positive in male homozygous for the *Lmr14* (D2Mit283) BALB/c allele (CC) (*R* = 0.51, *P* = 0.016) and STS allele (SS) (*R* = 0.50, *P* = 0.00088), but no correlation was observed in heterozygotes (*R* = −0.013, *P* = 0.92).

## Discussion

### Eosinophil infiltration in strain CcS-9 exceeds that of both parents

Strain CcS-9 that contains a set of approximately 12.5% genes of the donor strain STS and 87.5% genes of the background strain BALB/c exhibited numbers of infiltrating eosinophils (Fig. 1, Table 1) exceeding those in both parental strains BALB/c and STS. The observations of progeny having a phenotype, which is beyond the range of the phenotype of its parents, are not rare in traits controlled by multiple genes. It was detected in different tests of immune responses of RC strains in vitro [51–56] and in vivo [21, 57–60], and in analysis of expression quantitative trait loci (QTLs) from the livers of chromosome substitution strains [61]. These observations are due to multiple gene-gene interactions of QTLs, which in new combinations of these genes in RC or chromosomal substitution strains can lead to the appearance of new phenotypes that exceed their range in parental strains. In addition, with traits controlled by multiple loci, parental strains often contain eosinophil high infiltration alleles at some of them and eosinophil low infiltration alleles at others, and some progeny may receive predominantly eosinophil high infiltration alleles from both parents.

### Sex influence on eosinophil infiltration

Our data show a sex influence on eosinophil numbers in the inguinal lymph nodes. Differences between the immune system of females and males have been well documented [62–64] and could result in differences in susceptibility to diseases with immune component. Immune responses including those involving eosinophils might be modulated by steroid hormones [65, 66]. Moreover, some of the differences between females and males might be due to sex-specific genetic architecture, characterized by profound gene-sex interactions [67, 68]. This would mean that some genes controlling response to *L. major* might operate differently in the two sexes. Indeed, locus *Lmr14* controls eosinophil infiltration only in males. Genes controlling infections that appear to be sex dependent have been observed also with other infectious agents such as viruses [69–71], bacteria [72], parasites [58], and fungi [73] and helminths [74]. Some of sex-dependent QTLs exhibit a higher or exclusive influence on susceptibility in females [58, 69, 71–73] or males [69, 71–74], phenotypic effect of other genes is present in both sexes, but with opposite direction of effect [69, 70]. All these reported loci are situated on autosomal chromosomes. In contrast to the sex chromosomes, the autosomal genome is shared by both sexes. However, although the DNA sequence, gene structure, and frequency of polymorphism on the autosomes do not differ between males and females, the regulatory genome is sexually dimorphic [68].

Future genetic and functional studies will help to establish the mechanistic basis of the observed gene-sex interactions.

### Loci controlling eosinophil infiltration and other immune traits

The *Lmr* loci influencing eosinophil infiltration may be related to QTLs that determine certain immunologically relevant traits, because they co-map with other immunological functional polymorphisms.

Interestingly, two of the eosinophil controlling loci, *Lmr15* and *Lmr26* co-localize with loci that determine hemopoietic cell cycling measured by cobblestone area-forming cell (CAFC) assay using cells from the bone marrow [75]. *Lmr15* encompasses the mouse ortholog of human gene *IL5*, whose polymorphism was found to be associated with eosinophil counts in the blood [76], and *Lmr26* co-localizes also with locus *Tria5* that modifies in vitro proliferation of mouse splenocytes stimulated by soluble anti-CD3 [77].

**Fig. 3:**
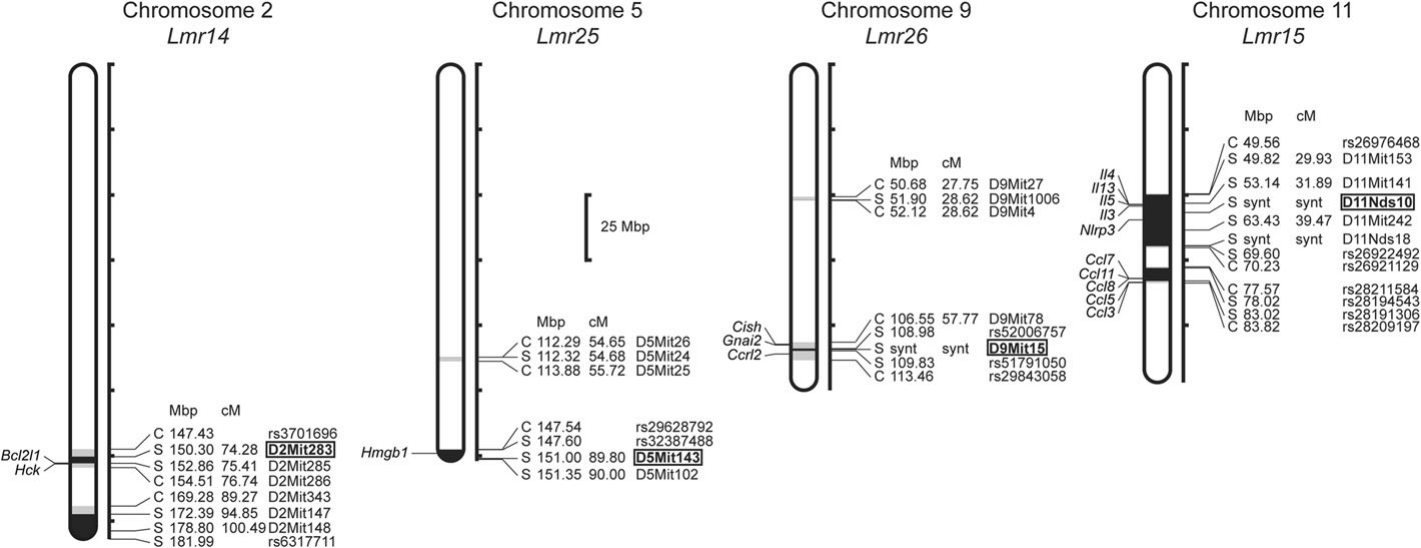
Loci controlling eosinophil numbers in CcS-9. The regions of STS and BALB/c origins are represented as *dark* and *white*, respectively; the boundary regions of undetermined origin are *shaded. C* and *S* indicate the presence of BALB/c and STS allele, respectively. Only the markers and SNPs defining the boundaries the STS-derived segment and the markers that were tested for linkage are shown. The markers that exhibit significant *P* values (genome-wide corrected) are shown in *bold. Lmr* loci on chromosomes 2 and 11 detected in a cross between CcS-9 and BALB/c overlap with loci *Lmr14* [91–93] and *Lmr15* [91, 93] detected in cross of CcS-16 with BALB/c, and were therefore given the same name. Abbreviations show genes that have been reported to be involved in eosinophil functions: *Bcl2l1* (BCL2-like 1) [78], *Ccl11* (chemokine (C-C motif) ligand 11) [79] *Ccl3* (chemokine (C-C motif) ligand 3) [79], *Ccl5* (chemokine (C-C motif) ligand 5) [79], *Ccl7* (chemokine (C-C motif) ligand 7) [79], *Ccl8* (chemokine (C-C motif) ligand 8) [80], *Ccrl2* (chemokine (C-C motif) receptor-like 2) [81], *Cish* (cytokine inducible SH2-containing protein) [82], *Gnai2* (guanine nucleotide binding protein (G protein), alpha inhibiting 2) [83], *Hck* (hemopoietic cell kinase) [84], *Hmgb1* (high mobility group box 1) [85], *Il13* (interleukin 13) [86], *Il3* (interleukin 3) [87], *Il4* (interleukin 4) [88], *Il5* (interleukin 5) [89], and *Nlrp3* (NLR family, pyrin domain containing 3) [90]. However, the effects of eosinophil controlling loci might be caused by genes that are at the present not considered as candidates

The four described loci comprise several genes (Fig. 3), whose biological function is compatible with the effects on eosinophil infiltration [78–90] and their potential role can now be investigated. However, the effects of these *Lmr* loci might be also caused by genes that are at the present not considered as candidates. The issue of identity of eosinophil controlling genes and their possible relationship to other immune traits will be resolved by a recombinational analysis.

The positive correlation between eosinophil infiltration and parasite load suggests that the observed eosinophilic infiltration reflects ineffective inflammation. This is in agreement with kinetic studies showing that parasite presence preceded presence of infiltrating cells including eosinophils. This infiltration was higher in mice that were unable to control infection [41].

We have found positive correlation between eosinophil infiltration and parasite numbers in *Lmr14* in homozygous (CC or SS), but not in heterozygous (CS) F_2_ hybrid males. The lack of positive correlation between eosinophil infiltration and parasite load in *Lmr14* heterozygotes (CS) may reflect a more effective inflammation process, perhaps facilitated by other phenotypic effects of *Lmr14* that include circulating levels of IFNγ, TNF, IgE, and IL-12 [91] and possibly other as yet undetected regulatory effects. This possibility has to be tested in future experiments.

## Conclusions

This is the first demonstration of genetic loci and sex influence controlling infiltration of eosinophils into the lymph nodes and its relationship with parasite load. Some of these loci comprise genes with broader biological and immunological effects, so they might be relevant also in control of other diseases and symptoms mediated by eosinophils.

Our data also suggest that ignoring sex in gene mapping might prevent detection of sex-dependent QTLs.

## Abbreviations

CcS/Dem: Series of recombinant congenic strains derived from the mouse donor strain STS/A (STS) and the background strain BALB/cHeA (BALB/c)
QTL: Quantitative trait locus
RCS: Recombinant congenic strains

## References

[1] Vom Steeg LG, Klein SL (2016). SeXX matters in infectious disease pathogenesis. PLoS Pathog.

[2] Mukhopadhyay D, Mukherjee S, Ghosh S, Roy S, Saha B, Das NK, Chatterjee M (2016). A male preponderance in patients with Indian post kala-azar dermal leishmaniasis is associated with increased circulating levels of testosterone. Int J Dermatol.

[3] Alvar J, Velez ID, Bern C, Herrero M, Desjeux P (2012). Leishmaniasis worldwide and global estimates of its incidence. PLoS One.

[4] GBD 2013 DALYs and HALE Collaborators (2015). Global, regional, and national disability-adjusted life years (DALYs) for 306 diseases and injuries and healthy life expectancy (HALE) for 188 countries, 1990-2013: quantifying the epidemiological transition. Lancet.

[5] Lipoldová M, Demant P (2006). Genetic susceptibility to infectious disease: lessons from mouse models of leishmaniasis. Nat Rev Genet.

[6] Noronha FS, Cruz JS, Beirão PS, Horta MF (2000). Macrophage damage by *Leishmania amazonensis* cytolysin: evidence of pore formation on cell membrane. Infect Immun.

[7] Rodríguez NE, Wilson ME (2014). Eosinophils and mast cells in leishmaniasis. Immunol Res.

[8] Klein SL (2004). Hormonal and immunological mechanisms mediating sex differences in parasite infection. Parasite Immunol.

[9] Snider H, Lezama-Davila C, Alexander J, Satoskar AR (2009). Sex hormones and modulation of immunity against leishmaniasis. Neuroimmunomodulation.

[10] Alexander J, Irving K, Snider H, Satoskar A, Klein SL, Roberts CW (2010). Sex hormones of host responses against parasites. Sex hormons and immunity to infection.

[11] Roberts CW, Walker W, Alexander J (2001). Sex-associated hormones and immunity to protozoan parasites. Clin Microbiol Rev.

[12] Bernin H, Lotter H (2014). Sex bias in the outcome of human tropical infectious diseases: influence of steroid hormones. J Infect Dis.

[13] Al-Jawabreh A, Dumaidi K, Ereqat S, Al-Jawabreh H, Nasereddin A, Azmi K, Barghuthy F, Sawalha S, Salah I, Abdeen Z. Molecular epidemiology of human cutaneous leishmaniasis in Jericho and its vicinity in Palestine from 1994 to 2015. Infect Genet Evol. 2016. doi: 10.1016/j.meegid.2016.06.007. [Epub ahead of print]. 10.1016/j.meegid.2016.06.007 27268151

[14] Armijos RX, Weigel MM, Izurieta R, Racines J, Zurita C, Herrera W, Vega M (1997). The epidemiology of cutaneous leishmaniasis in subtropical Ecuador. Trop Med Int Health.

[15] Guerra-Silveira F, Abad-Franch F (2013). Sex bias in infectious disease epidemiology: patterns and processes. PLoS One.

[16] Khosravani M, Moemenbellah-Fard MD, Sharafi M, Rafat-Panah A (2016). Epidemiologic profile of oriental sore caused by *Leishmania* parasites in a new endemic focus of cutaneous leishmaniasis, southern Iran. J Parasit Dis.

[17] Travi BL, Osorio Y, Melby PC, Chandrasekar B, Arteaga L, Saravia NG (2002). Gender is a major determinant of the clinical evolution and immune response in hamsters infected with *Leishmania* spp. Infect Immun.

[18] Mock BA, Nacy CA (1988). Hormonal modulation of sex differences in resistance to *Leishmania major* systemic infections. Infect Immun.

[19] Mock BA, Fortier AH, Potter M, Nacy CA (1985). Genetic control of systemic *Leishmania major* infections: dissociation of intrahepatic amastigote replication from control by the Lsh gene. Infect Immun.

[20] Alexander J (1988). Sex differences and cross-immunity in DBA/2 mice infected with *L. mexicana* and *L. major*. Parasitology.

[21] Kobets T, Havelková H, Grekov I, Volkova V, Vojtíšková J, Slapničková M, Kurey I, Sohrabi Y, Svobodová M, Demant P, Lipoldová M (2012). Genetics of host response to *Leishmania tropica* in mice—different control of skin pathology, chemokine reaction, and invasion into spleen and liver. PLoS Negl Trop Dis.

[22] Giannini MSH (1986). Sex-influenced response in the pathogenesis of cutaneous leishmaniasis in mice. Parasite Immunol.

[23] Kobets T, Grekov I, Lipoldová M (2012). Leishmaniasis: prevention, parasite detection and treatment. Curr Med Chem.

[24] Kurey I, Kobets T, Havelková H, Slapničková M, Quan L, Trtková K, Grekov I, Svobodová M, Stassen AP, Hutson A, Demant P, Lipoldová M (2009). Distinct genetic control of parasite elimination, dissemination, and disease after *Leishmania major* infection. Immunogenetics.

[25] Rosenberg HF, Dyer KD, Foster PS (2013). Eosinophils: changing perspectives in health and disease. Nat Rev Immunol.

[26] Akuthota P, Wang HB, Spencer LA, Weller PF (2008). Immunoregulatory roles of eosinophils: a new look at a familiar cell. Clin Exp Allergy.

[27] Akuthota P, Weller PF (2015). Spectrum of eosinophilic end-organ manifestations. Immunol Allergy Clin North Am.

[28] Blanchard C, Rothenberg ME (2009). Biology of the eosinophil. Adv Immunol.

[29] Gusareva ES, Kurey I, Grekov I, Lipoldová M (2014). Genetic regulation of immunoglobulin E level in different pathological states: integration of mouse and human genetics. Biol Rev Camb Philos Soc.

[30] Raap U, Wardlaw AJ (2008). A new paradigm of eosinophil granulocytes: neuroimmune interactions. Exp Dermatol.

[31] Percopo CM, Dyer KD, Ochkur SI, Luo JL, Fischer ER, Lee JJ, Lee NA, Domachowske JB, Rosenberg HF (2014). Activated mouse eosinophils protect against lethal respiratory virus infection. Blood.

[32] Sasaki O, Sugaya H, Ishida K, Yoshimura K (1993). Ablation of eosinophils with anti-IL-5 antibody enhances the survival of intracranial worms of *Angiostrongylus cantonensis* in the mouse. Parasite Immunol.

[33] Shin EH, Osada Y, Chai JY, Matsumoto N, Takatsu K, Kojima S (1997). Protective roles of eosinophils in *Nippostrongylus brasiliensis* infection. Int Arch Allergy Immunol.

[34] Martin C, Le Goff L, Ungeheuer MN, Vuong PN, Bain O (2000). Drastic reduction of a filarial infection in eosinophilic interleukin-5 transgenic mice. Infect Immun.

[35] Ramalingam T, Porte P, Lee J, Rajan TV (2005). Eosinophils, but not eosinophil peroxidase or major basic protein, are important for host protection in experimental *Brugia pahangi* infection. Infect Immun.

[36] Swartz JM, Dyer KD, Cheever AW, Ramalingam T, Pesnicak L, Domachowske JB, Lee JJ, Lee NA, Foster PS, Wynn TA, Rosenberg HF (2006). *Schistosoma mansoni* infection in eosinophil lineage-ablated mice. Blood.

[37] Fabre V, Beiting DP, Bliss SK, Gebreselassie NG, Gagliardo LF, Lee NA, Lee JJ, Appleton JA (2009). Eosinophil deficiency compromises parasite survival in chronic nematode infection. J Immunol.

[38] Huang L, Gebreselassie NG, Gagliardo LF1, Ruyechan MC, Luber KL, Lee NA, Lee JJ, Appleton JA (2015). Eosinophils mediate protective immunity against secondary nematode infection. J Immunol.

[39] Oliveira SH, Fonseca SG, Romão PR, Figueiredo F, Ferreira SH, Cunha FQ (1998). Microbicidal activity of eosinophils is associated with activation of the arginine-NO pathway. Parasite Immunol.

[40] Beil WJ, Meinardus-Hager G, Neugebauer DC, Sorg C (1992). Differences in the onset of the inflammatory response to cutaneous leishmaniasis in resistant and susceptible mice. J Leukoc Biol.

[41] Belkaid Y, Mendez S, Lira R, Kadambi N, Milon G, Sacks D (2000). A natural model of *Leishmania major* infection reveals a prolonged “silent” phase of parasite amplification in the skin before the onset of lesion formation and immunity. J Immunol.

[42] Demant P, Hart AAM (1996). Recombinant congenic strains—a new tool for analysing genetic traits determined by more than one gene. Immunogenetics.

[43] Grekov I, Svobodová M, Nohýnková E, Lipoldová M (2011). Preparation of highly infective *Leishmania* promastigotes by cultivation on SNB-9 biphasic medium. J Microbiol Meth.

[44] Lipoldová M, Svobodová M, Krulová M, Havelková H, Badalová J, Nohýnková E, Holáň V, Hart AAM, Volf P, Demant P (2000). Susceptibility to *Leishmania major* infection in mice: multiple loci and heterogeneity of immunopathological phenotypes. Genes Immun.

[45] Stassen AP, Groot PC, Eppig JT, Demant P (1996). Genetic composition of the recombinant congenic strains. Mamm Genome.

[46] Sohrabi Y, Havelková H, Kobets T, Šíma M, Volkova V, Grekov I, Jarošíková T, Kurey I, Vojtíšková J, Svobodová M, Demant P, Lipoldová M (2013). Mapping the genes for susceptibility and response to *Leishmania tropica* in mouse. PLoS Negl Trop Dis.

[47] Šíma M, Kocandová L, Lipoldová M. Genotyping of short tandem repeats (STRs) markers with 6 bp or higher length difference using PCR and high resolution agarose electrophoresis. Protoc Exch. 2015. doi:10.1038/protex.2015.054.

[48] Krulová M, Havelková H, Kosařová M, Holáň V, Hart AA, Demant P (1997). IL-2-induced proliferative response is controlled by loci *Cinda1* and *Cinda2* on mouse chromosomes 11 and 12: a distinct control of the response induced by different IL-2 concentration. Genomics.

[49] Kobets T, Badalová J, Grekov I, Havelková H, Svobodová M, Lipoldová M (2010). *Leishmania* parasite detection and quantification using PCR-ELISA. Nat Protoc.

[50] Lander ES, Schork NJ (1994). Genetic dissection of complex traits. Science.

[51] Lipoldová M, Kosařová M, Zajícová A, Holáň V, Hart AA (1995). Separation of multiple genes controlling the T-cell proliferative response to IL-2 and anti-CD3 using recombinant congenic strains. Immunogenetics.

[52] Holáň V, Lipoldová M, Demant P (1996). Identical genetic control of MLC reactivity to different MHC incompatibilities, independent of production of and response to IL-2. Immunogenetics.

[53] Havelková H, Badalová J, Demant P, Lipoldová M (2000). A new type of genetic regulation of allogeneic response. A novel locus on mouse chromosome 4, *Alan2* controls MLC reactivity to three different alloantigens: C57BL/10, BALB/c and CBA. Genes Immun.

[54] Lipoldová M, Havelková H, Badalová J, Demant P (2005). Novel loci controlling lymphocyte proliferative response to cytokines and their clustering with loci controlling autoimmune reactions, macrophage function and lung tumor susceptibility. Int J Cancer.

[55] Havelková H, Holáň V, Kárník I, Lipoldová M (2006). Mouse model for analysis of non-MHC genes that influence allogeneic response: recombinant congenic strains of OcB/Dem series that carry identical *H2* locus. Cent Eur J Biol.

[56] Lipoldová M, Havelková H, Badalová J, Vojtíšková J, Quan L, Krulová M, Sohrabi Y, Stassen AP, Demant P. Loci controlling lymphocyte production of interferon γ after alloantigen stimulation *in vitro *and their co-localization with genes controlling lymphocyte infiltration of tumors and tumor susceptibility. Cancer Immunol Immunother. 2010;59:203–13. 10.1007/s00262-009-0739-y PMC2776939 19655140

[57] Lipoldová M, Svobodová M, Havelková H, Krulová M, Badalová J (2002). Mouse genetic model for clinical and immunological heterogeneity of leishmaniasis. Immunogenetics.

[58] Šíma M, Havelková H, Quan L, Svobodová M, Jarošíková T, Vojtíšková J, Stassen APM, Demant P, Lipoldová M. Genetic control of resistance to *Trypanosoma brucei brucei*infection in mice. PLoS Negl Trop Dis. 2011;5:e1173. 10.1371/journal.pntd.0001173 PMC3110168 21666791

[59] Szymanska H, Sitarz M, Krysiak E, Piskorowska J, Czarnomska A, Skurzak H, Hart AA, de Jong D, Demant P (1999). Genetics of susceptibility to radiation-induced lymphomas, leukemias and lung tumors studied in recombinant congenic strains. Int J Cancer.

[60] Palus M, Vojtíšková J, Salát J, Kopecký J, Grubhoffer L, Lipoldová M, Demant P, Růžek D (2013). Mice with different susceptibility to tick-borne encephalitis virus infection show selective neutralizing antibody response and inflammatory reaction in the central nervous system. J Neuroinflamm.

[61] Shockley KR, Churchill GA (2006). Gene expression analysis of mouse chromosome substitution strains. Mamm Genome.

[62] Halberg F, Hamerston O, Bittner JJ (1957). Sex difference in eosinophil counts in tall blood of mature B1 mice. Science.

[63] Madalli S, Beyrau M, Whiteford J, Duchene J, Singh Nandhra I, Patel NS, Motwani MP, Gilroy DW, Thiemermann C, Nourshargh S, Scotland RS (2015). Sex-specific regulation of chemokine Cxcl5/6 controls neutrophil recruitment and tissue injury in acute inflammatory states. Biol Sex Differ.

[64] Case LK, Teuscher C (2015). Y genetic variation and phenotypic diversity in health and disease. Biol Sex Differ.

[65] Hamano N, Terada N, Maesako K, Numata T, Konno A (1998). Effect of sex hormones on eosinophilic inflammation in nasal mucosa. Allergy Asthma Proc.

[66] Keselman A, Heller N (2015). Estrogen signaling modulates allergic inflammation and contributes to sex differences in asthma. Front Immunol.

[67] Bhasin JM, Chakrabarti E, Peng DQ, Kulkarni A, Chen X, Smith JD (2008). Sex specific gene regulation and expression QTLs in mouse macrophages from a strain intercross. PLoS One.

[68] Ober C, Loisel DA, Gilad Y (2008). Sex-specific genetic architecture of human disease. Nat Rev Genet.

[69] Butterfield RJ, Roper RJ, Rhein DM, Melvold RW, Haynes L (2003). Sex-specific quantitative trait loci govern susceptibility to Theiler’s murine encephalomyelitis virus-induced demyelination. Genetics.

[70] Schuurhof A, Bont L, Siezen CL, Hodemaekers H, van Houwelingen HC (2010). Interleukin-9 polymorphism in infants with respiratory syncytial virus infection: an opposite effect in boys and girls. Pediatr Pulmonol.

[71] Boivin GA, Pothlichet J, Skamene E, Brown EG, Loredo-Osti JC, Sladek R, Vidal SM (2012). Mapping of clinical and expression quantitative trait loci in a sex-dependent effect of host susceptibility to mouse-adapted influenza H3N2/HK/1/68. J Immunol.

[72] Min-Oo G, Lindqvist L, Vaglenov A, Wang C, Fortin P (2008). Genetic control of susceptibility to pulmonary infection with *Chlamydia pneumoniae* in the mouse. Genes Immun.

[73] Carroll SF, Loredo Osti JC, Guillot L, Morgan K, Qureshi ST (2008). Sex differences in the genetic architecture of susceptibility to *Cryptococcus neoformans* pulmonary infection. Genes Immun.

[74] Hayes KS, Hager R, Grencis RK (2014). Sex-dependent genetic effects on immune responses to a parasitic nematode. BMC Genomics.

[75] De Haan G, Van Zant G (1999). Genetic analysis of hemopoietic cell cycling in mice suggests its involvement in organismal life span. FASEB J.

[76] Gudbjartsson DF (2009). Sequence variants affecting eosinophil numbers associate with asthma and myocardial infarction. Nat Genet.

[77] Havelková H, Kosařová M, Krulová M, Demant P, Lipoldová M (1999). T-cell proliferative response is controlled by loci *Tria4* and *Tria5* on mouse chromosomes 7 and 9. Mamm Genome.

[78] Dibbert B, Daigle I, Braun D, Schranz C, Weber M, Blaser K, Zangemeister-Wittke U, Akbar AN, Simon HU (1998). Role for Bcl-xL in delayed eosinophil apoptosis mediated by granulocyte-macrophage colony-stimulating factor and interleukin-5. Blood.

[79] Simson L, Foster PS (2000). Chemokine and cytokine cooperativity: eosinophil migration in the asthmatic response. Immunol Cell Biol.

[80] Islam SA, Chang DS, Colvin RA, Byrne MH, McCully ML, Moser B, Lira SA, Charo IF, Luster AD (2011). Mouse CCL8, a CCR8 agonist, promotes atopic dermatitis by recruiting IL-5+ T(H)2 cells. Nat Immunol.

[81] Otero K, Vecchi A, Hirsch E, Kearley J, Vermi W, Del Prete A, Gonzalvo-Feo S, Garlanda C, Azzolino O, Salogni L, Lloyd CM, Facchetti F, Mantovani A, Sozzani S (2010). Nonredundant role of CCRL2 in lung dendritic cell trafficking. Blood.

[82] Yang XO, Zhang H, Kim BS, Niu X, Peng J, Chen Y, Kerketta R, Lee YH, Chang SH, Corry DB, Wang D, Watowich SS, Dong C (2013). The signaling suppressor CIS controls proallergic T cell development and allergic airway inflammation. Nat Immunol.

[83] Pero RS, Borchers MT, Spicher K, Ochkur SI, Sikora L, Rao SP, Abdala-Valencia H, O'Neill KR, Shen H, McGarry MP, Lee NA, Cook-Mills JM, Sriramarao P, Simon MI, Birnbaumer L, Lee JJ (2007). Galphai2-mediated signaling events in the endothelium are involved in controlling leukocyte extravasation. Proc Natl Acad Sci U S A.

[84] El-Shazly A, Yamaguchi N, Masuyama K, Suda T, Ishikawa T (1999). Novel association of the src family kinases, hck and c-fgr, with CCR3 receptor stimulation: a possible mechanism for eotaxin-induced human eosinophil chemotaxis. Biochem Biophys Res Commun.

[85] Lotfi R, Lee JJ, Lotze MT (2007). Eosinophilic granulocytes and damage-associated molecular pattern molecules (DAMPs): role in the inflammatory response within tumors. J Immunother.

[86] Pope SM, Brandt EB, Mishra A, Hogan SP, Zimmermann N, Matthaei KI, Foster PS, Rothenberg ME (2001). IL-13 induces eosinophil recruitment into the lung by an IL-5- and eotaxin-dependent mechanism. J Allergy Clin Immunol.

[87] Håkansson L, Venge P (1994). Priming of eosinophil and neutrophil migratory responses by interleukin 3 and interleukin 5. APMIS.

[88] Brusselle GG, Kips JC, Tavernier JH, van der Heyden JG, Cuvelier CA, Pauwels RA, Bluethmann H (1994). Attenuation of allergic airway inflammation in IL-4 deficient mice. Clin Exp Allergy.

[89] Kopf M, Brombacher F, Hodgkin PD, Ramsay AJ, Milbourne EA, Dai WJ, Ovington KS, Behm CA, Köhler G, Young IG, Matthaei KI (1996). IL-5-deficient mice have a developmental defect in CD5+ B-1 cells and lack eosinophilia but have normal antibody and cytotoxic T cell responses. Immunity.

[90] Kvarnhammar AM, Petterson T, Cardell LO (2011). NOD-like receptors and RIG-I-like receptors in human eosinophils: activation by NOD1 and NOD2 agonists. Immunology.

[91] Havelková H, Badalová J, Svobodová M, Vojtíšková J, Kurey I, Vladimirov V, Demant P, Lipoldová M (2006). Genetics of susceptibility to leishmaniasis in mice: four novel loci and functional heterogeneity of gene effects. Genes Immun.

[92] Badalová J, Svobodová M, Havelková H, Vladimirov V, Vojtíšková J, Engová J, Pilčík T, Volf P, Demant P, Lipoldová M (2002). Separation and mapping of multiple genes that control IgE level in *Leishmania major* infected mice. Genes Immun.

[93] Vladimirov V, Badalová J, Svobodová M, Havelková H, Hart AAM, Blažková H, Demant P, Lipoldová M (2003). Different genetic control of cutaneous and visceral disease after *Leishmania major* infection in mice. Infect Immun.

